# Mood Worsening on Days with High Pollen Counts is associated with a Summer Pattern of Seasonality

**DOI:** 10.1515/pteridines-2019-0016

**Published:** 2019-08-23

**Authors:** Faisal Akram, Tyler B. Jennings, John W. Stiller, Christopher A. Lowry, Teodor T. Postolache

**Affiliations:** Mood and Anxiety Program, University of Maryland School of Medicine, Baltimore, MD, 21201, USA; Saint Elizabeths Hospital, Psychiatry Residency Training Program, Washington, DC, 20032, USA; Mood and Anxiety Program, University of Maryland School of Medicine, Baltimore, MD, 21201, USA; Mood and Anxiety Program, University of Maryland School of Medicine, Baltimore, MD, 21201, USA; Saint Elizabeths Hospital, Psychiatry Residency Training Program, Washington, DC, 20032, USA; Maryland State Athletic Commission, Baltimore, MD, USA; Department of Integrative Physiology, Center for Neuroscience, and Center for Microbial Exploration, University of Colorado Boulder, Boulder, CO 80309, USA; Department of Physical Medicine & Rehabilitation, University of Colorado Anschutz Medical Campus, Aurora, CO 80045, USA; Veterans Health Administration, Rocky Mountain Mental Illness Research Education and Clinical Center (MIRECC), Rocky Mountain Regional Veterans Affairs Medical Center (RMRVAMC), Aurora, CO, 80045, USA; Military and Veteran Microbiome: Consortium for Research and Education (MVM-CoRE), Aurora, CO 80045, USA; Mood and Anxiety Program, University of Maryland School of Medicine, Baltimore, MD, 21201, USA; Saint Elizabeths Hospital, Psychiatry Residency Training Program, Washington, DC, 20032, USA; Department of Physical Medicine & Rehabilitation, University of Colorado Anschutz Medical Campus, Aurora, CO 80045, USA; Veterans Health Administration, Rocky Mountain Mental Illness Research Education and Clinical Center (MIRECC), Rocky Mountain Regional Veterans Affairs Medical Center (RMRVAMC), Aurora, CO, 80045, USA; Amish Research Clinic of the University of Maryland, Lancaster, PA, 17602, USA

**Keywords:** Aeroallergen, Mood Disorder, Old Order Amish, Seasonality, Summer SAD

## Abstract

**Background:**

Summer/spring-type seasonal affective disorder (S-SAD) is the less common subtype of seasonal affective disorder and evidence regarding potential triggers of S-SAD is scarce. Recent reports support association of airborne-pollen with seasonal exacerbation of depression (mood seasonality) and timing of suicidal behavior. Therefore, we hypothesized that Old Order Amish (OOA) with summer/spring pattern of seasonality (abbreviated as summer pattern) and S-SAD will have significant mood worsening on high pollen days.

**Methods:**

A seasonal pattern of mood worsening and SAD parameters were estimated using Seasonal Pattern Assessment Questionnaire (SPAQ). Age- and gender-adjusted ANCOVAs and post hoc analyses were conducted to compare mood worsening on days with high pollen counts between summer-pattern vs no-summer-pattern of mood worsening, S-SAD vs no-S-SAD, winter-pattern vs no-winter-pattern of mood worsening, and W-SAD vs no-W-SAD groups.

**Results:**

The prevalence of S-SAD was 0.4%, while 4.5% of individuals had a summer pattern of mood seasonality. A statistically significant difference for mood worsening on high pollen days was observed between summer-pattern vs no-summer-pattern of mood worsening (*p* = 0.006). The significant association between S-SAD vs no-SAD groups (*p* = 0.032) for mood worsening on high pollen days did not withstand Bonferroni adjustment for multiple comparisons. No significant association was found for winter-pattern vs no-winter-pattern of mood worsening (*p* = 0.61) and for W-SAD vs no-W-SAD (*p* = 0.19) groups.

**Conclusion:**

Our results are consistent with previous studies implicating links between aeroallergen exposure and summer pattern of seasonality, but not the winter pattern of seasonality.

## Introduction

Seasonal changes in mood, behavior, and neurovegetative functions have been well characterized and reflect interactions between biological systems and environment [1, 2]. These changes have been extensively studied in relation to affective disorders and a distinct phenotype (seasonal subtype), characterized by onset of mood symptoms in one season and spontaneous remission in other seasons, has been identified [3–8]. Seasonal affective disorder (SAD), coded as a specifier in DSM-5 [9], is associated with significant morbidity [10, 11] and impairment in social [12], occupational [13], and cognitive functioning [14]. Epidemiological studies show that the lifetime prevalence of SAD among the general population lies between 0.4–2.9% and most depressive episodes occur in the fall or winter season [15, 16]. In addition to the more prevalent fall/winter-type (W-SAD), a summer/spring-type (S-SAD) has also been characterized in which individuals experience depression in summer or spring [17–20]. Wehr et al. (1991) first contrasted the dominant clinical presentation of the depressive episodes in S-SAD (decreased appetite and insomnia) and W-SAD (predominant atypical features, with increased appetite, weight gain, carbohydrate craving and hypersomnia) [20]. Both forms of depressive episodes have similarly high functional impairment, decreased sexual interest, elevated fatigue, and social avoidance, and comorbid anxiety.

S-SAD is more prevalent in tropical regions as compared to temperate regions [18, 21–23]. Morissey et al. (1996) reported that 9.2% of respondents met criteria for S-SAD as compared to 1.7% meeting criteria for W-SAD in northern Australia [18]. In another survey undertaken in the northern tropics, prevalence of S-SAD was 6.19% as compared to 1.03% for W-SAD [24]. Although W-SAD predominates in temperate regions, a small proportion of individuals also have a summer pattern of seasonal affective changes. An epidemiological survey done in four different latitudes of the USA reported that the prevalence of S-SAD was 0.5% in Nashua, NH, 3.1% in New York, NY, 1% in Montgomery County, MD and 1.2% in Sarasota, FL [19].

Although W-SAD has been hypothesized to be triggered by shortened photoperiods and decreased sunlight exposure in winter in vulnerable individuals, evidence regarding etiology of S-SAD has been scarce. Wehr et al. suggested that heat exposure, in contrast to light, may lead to S-SAD in individuals with heat vulnerability, possibly a result of thermoregulatory dysregulation [17]. Similarly, Morrissey et al. (1996) reported that heat and humidity were the two most common environmental variables attributed to mood worsening by respondents who met criteria for S-SAD [18, 25]. Divergent results have also been reported. For instance, Soriano et al. (2007) showed that S-SAD was more prevalent in Romanian individuals with access to air conditioners [26], inconsistent with the hypothesis of heat exposure leading to S-SAD. An alternative explanation may be that spending more time indoors during summer may reduce light exposure, exercise, and social activities, resulting in mood worsening [26]. Other environmental variables suggested to be implicated in seasonal exacerbation of depression and seasonal peaks of suicide include airborne infectious agents [27], air pollution [28], ambient particulate exposure [29], and increased pollen exposure [30–32], and these agents are believed to mediate their effects through neuroinflammation. For example, allergic rhinitis, which has a strong seasonal component, is associated with depression in many cross-sectional studies [33–35]. Postolache et al. (2007) reported that allergic symptoms and depression scores were correlated in patients with recurrent mood disorders [36]. Another study by Chen et al. (2013) has shown that allergic rhinitis in adolescence is associated with depression in late adolescence and early adulthood [37]. Allergic rhinitis has also been shown to increase the risk of bipolar disorder among adolescents [38].

In North America, there are three distinct plant-based aeroallergen seasons: 1) tree pollen in spring; 2) grass pollen in summer; and 3) weed pollen in late summer and fall [39, 40]. The spring peaks of aeroallergens coincide with previously reported peaks in suicide rate [41, 42] and mood worsening (fall and summer) [31, 43] in various studies. Based on the suicide data from the General Mortality Database in a population of 37,824,174, Postolache et al. (2004) reported that tree pollen peaks were associated with non-violent suicide in women. This study was replicated by Stickley et al. (2017), who reported that pollen level of 30 to < 100 grains per cm^2^ was associated with an approximately 50% increased risk of suicide among women, but not men [44]. Similarly, in a large Danish study of 13,700 suicide events in a population of 2.86 million, Qin et al. (2012) noted a significant association between suicide risk and air pollen counts [32].

Aeroallergens elicit an immune response through activation of CD4+ T helper type 2 (Th2) cells and secretion of various cytokines including interleukin (IL)-4, IL-5, IL-6, IL-10, and IL-13 [45]. Subsequently, IL-4 from Th2 cells promotes B cell transformation to immunoglobulin-secreting plasma cells and class switching of immunoglobulin G (IgG) to immunoglobulin E (IgE) [46]. These IgE molecules circulate in the bloodstream and bind to high affinity Fc receptors (FcɛRI) on mast cells [47]. Upon exposure to the specific aeroallergen, mast cells degranulate and release chemical inflammatory mediators, including histamine, tryptase, kininogenase, and prostaglandins, thereby initiating the early reaction of allergic immune response [48]. The late reaction is mainly propagated by eosinophils, synthesized leukotrienes, and cytokines, which cause increased vascular permeability, contraction of local smooth muscles, and increased mucus secretion [49]. Apart from causing symptoms related to local tissues (e.g. skin and nasal mucosa), proinflammatory cytokines from the periphery are able to reach the CNS, and they have the capacity to influence neurotransmitter metabolism, neuroendocrine function [50], sleep [51], memory [52], emotions [53], and cognition [54]. For example, major depressive disorder has been associated with increased serum acute phase reactants and proinflammatory cytokines such as C-reactive protein (CRP), interleukin (IL)-6, IL-1β, and tumor necrosis factor (TNF) [55]. Tonelli et al (2008) also reported in a postmortem brain study that increased mRNA transcription of Th2 cytokines IL-4 and IL-13 was found in the orbitofrontal cortex of suicide victims [56].

A growing number of studies have shown an association between seasonal allergic rhinitis (SAR) and depression [57]. Trikojat et al. (2017) reported that during acute allergic inflammation, SAR patients experienced a significant increase in Beck Depression Inventory (BDI-) II scores as compared to non-allergic controls and asymptomatic patients with a history of SAR [58]. In addition, increases in BDI-II scores in SAR patients were significantly associated with IL-6 levels, as well as IL-6/IL-10 and IFNγ/IL-10 ratios [58]. Similarly, Manalai et al. (2012) measured allergen-specific IgE (a vulnerability marker) in patients with recurrent mood disorders and reported that pollen-specific IgE positivity was associated with worsening of depression scores in bipolar disorder patients during high pollen season [59].

Although allergen exposure has been linked to mood disorders and suicide, its role in S-SAD has been understudied. One study, specifically by Guzman et al. (2007) has reported an association of global seasonality score and non-winter SAD with mood worsening on days with high pollen counts in urban college students [31]. Here, we specifically hypothesized that in Old Order Amish, a predominantly agrarian population with higher exposure to seasonal aeroallergens through farming activities and lack of air conditioning (keeping windows open in the warmer weather), individuals with S-SAD, but not W-SAD, have greater mood sensitivity to high pollen counts.

## Methods

### Study population

We conducted a cross-sectional survey of seasonality of mood based on seasonal pattern assessment questionnaire (SPAQ) in the Old Order Amish residing in rural areas of Lancaster, PA, USA. The Old Order Amish are an agrarian population that do not use modern technologies, including network electric light at home, and spend more time outdoors, exposed to natural daylight [60]. Therefore, Amish represent a convenient population to study seasonality without the confounding influence of network electric light exposure and urbanization.

### Seasonal pattern assessment questionnaire (SPAQ)

The SPAQ is the questionnaire that permits the calculation of the quantitative seasonality measure, i.e., the global seasonality score (GSS), based on six parameters – sleep duration, social activity, mood, weight, appetite, and energy level – tested by 6 questions and rated on a scale of 0 to 4 reflecting “no change” to “extremely marked change” in each parameter with season [61]. Simply adding scores on each of those items gives the GSS score. Severity of the problem is measured on a 5-point rating scale ranging from none to disabling. Seasonal pattern was identified through participants’ answer to the question: “At what time of the year do you feel worst?” The fall-winter pattern (abbreviated as Winter pattern) was defined as October-March, and the spring-summer interval (abbreviated as Summer pattern) was defined as April-September. SAD cases are identified based on a positive fall/winter or spring/summer seasonal pattern, a total GSS ≥ 11, and a problem score representing moderate to disabling severity. In addition, participants were asked if they experienced mood changes (worsening or improvement) on high pollen days. The SPAQ has shown good test-retest reliability (GSS α = 0.87, *p* < 0.001; Problem Rating Scores (PRS) α = 0.79, *p* < 0.001) in the Old Order Amish [62].

### Procedure

We mailed the SPAQ to 2260 Amish individuals, aged 18 and older, who were previously enrolled in studies of cardiovascular, metabolic, and bone health conducted at the University of Maryland [63, 64]. Included in the mail was a letter having directions to complete the questionnaire and a $1 bill as a token of appreciation. The Institutional Review Board of the University of Maryland School of Medicine approved the study.

#### Ethical approval:

The research related to human use has been complied with all the relevant national regulations, institutional policies and in accordance the tenets of the Helsinki Declaration, and has been approved by Institutional Review Board of the University of Maryland School of Medicine

#### Informed consent:

Informed consent has been obtained from all individuals included in this study

### Statistical analyses

The calculated variables were age, sex, BMI, GSS, S-SAD, W-SAD, and seasonality patterns for summer and winter.

We compared mood sensitivity to pollen in those with a summer pattern of seasonality versus those without a summer pattern, and between those with S-SAD versus those without S-SAD using ANCOVAs with adjustment for age and gender. Similarly, we conducted ANCOVAs, with adjustment for age and gender, to compare mood sensitivity to pollen between those with a winter pattern of seasonality versus those without a winter pattern of seasonality and between those with winter SAD versus those with no-winter SAD. We followed up significant overall ANCOVA models with post hoc Tukey’s Honestly Significant Difference Tests. Data analysis was performed using SAS 14.2 (Cary, NC, USA), and statistical significance was set at a two-tailed *p*-value of 0.05. We then applied a Bonferroni adjustment for multiple comparisons for the main results of the study.

## Results

The overall response rate was 57.8% as 1306 individuals, 736 women (56.3%) and 570 men (43.6%), completed the questionnaire. Mean age (± SEM) was 55.6 (± 14.8) years while mean GSS was 4.4 (± 3.4). The prevalence of S-SAD was 0.4%, while 60 individuals (4.5%) had a summer pattern of mood seasonality. There were 11 (0.84%) cases of W-SAD and 6.12% individuals had a winter pattern of mood seasonality.

An ANCOVA adjustment for age and gender yielded a statistically significant difference in mood changes on days with high pollen counts between summer-pattern and no-summer pattern groups [*F* (1,399) = 7.7, *p* = 0.006]. Tukey’s Honestly Significant Difference Test identified significant differences between the summer-pattern and no-pattern groups regarding the degree of mood changes on days with high pollen counts (*p* = 0.006).

Similarly, an ANCOVA, after adjustment for age and gender, was statistically significant for difference in mood worsening on days with high pollen counts between S-SAD and no-S-SAD [*F* (1,399) = 5.2, *p* = 0.023]. Congruent with our previous results, Tukey’s Honestly Significant Difference Test identified significant differences between the S-SAD and no-S-SAD groups regarding the degree of mood changes on days with high pollen counts (*p* = 0.032).

Age- and gender-adjusted ANCOVAs showed non-significant results on comparing mood worsening on days with high pollen counts between winter pattern vs no-winter pattern groups [*F* (1,399) = 0.2, *p* = 0.617], as well as between winter SAD vs no-winter SAD groups [*F* (1,399) =1.7, *p* = 0.196].

A linear regression analysis was used to estimate the relationship between participants’ reported mood worsening on high pollen days and their GSS scores, mean = 4.41 (SD + 3.47). Although we hypothesized a negative association between GSS and mood changes on high pollen days, no significant association (*p* = 0.19) was found for this regression (with and without adjustment for age and sex).

Applying a Bonferroni correction for multiple comparisons lowers the criterion α to 0.017. This maintained the significance of the association of summer pattern with mood worsening with high pollen counts, while rendering non-significant the association between S-SAD and mood worsening with high pollen counts.

## Discussion

The central finding of this study is the self-reported mood worsening on days with high pollen counts in the Old Order Amish with a spring/summer-type but not in those with a fall/winter-type pattern of seasonality. We also found that the association between self-reported mood worsening on days with high pollen counts and S-SAD was initially significant but did not hold significance after Bonferroni adjustment for multiple comparisons. Our findings are only partially consistent with the previous study by Guzman et al. (2007) who reported that mood sensitivity with a high pollen count is associated with a greater seasonality of mood and predicts SAD of non-winter type [31]. It remains to be explored whether pollen-specific allergy has a causal effect on the spring/summer pattern of seasonality, or if that relationship is driven by a “hidden variable”. Nevertheless, this study lends further support to the inflammatory hypothesis of depressive disorders [65, 66], with a specific focus on persistent immune activation and increased brain expression levels of allergy-related Th2 cytokines such as IL-4, IL-5, and IL-13 [56], as well as Th1 cytokines (i.e., tumor necrosis factor (TNF)-alpha, IL-6), and decreased levels of regulatory cytokines such as IL-10 [55, 67, 68]. Allergic inflammation is triggered in a robust seasonal pattern by seasonal aeroallergens, such as tree pollen in spring [39]. In addition, neuroinflammation in allergic rhinitis may be mediated through direct passage of inflammatory signals from the nasal cavities to the brain [27]. For example, intra-nasal allergic sensitization and re-exposure in rodents results in anxiety and impaired social interaction, as well as an increase in Th2-mediated cytokines in the brain [69].

Although the exact mechanisms through which allergen-induced cytokines and inflammatory mediators lead to depression remain to be fully elucidated, converging lines of evidence point to alterations in tryptophan and kynurenine metabolism [70]. Tryptophan is an essential amino acid that is not only a precursor of serotonin [71] but is also metabolized to kynurenine through the kynurenine pathway of tryptophan metabolism [72]. The kynurenine pathway is mainly regulated by the enzyme indoleamine-2,3-dioxygenase-1 (IDO-1), which is activated principally via Th1 responses, especially, IFNγ [73, 74]. As a result, conditions with Th1 immune activation such as cancers, viral infections, or IFNγ therapy lead to lowered serum tryptophan levels, increased kynurenine/tryptophan ratio, and possibly a shift in the balance from serotonin to kynurenine biosynthesis [75, 76]. During the acute phase of allergic response, aeroallergens induce predominantly a Th2 response and decrease Th1 immune activation [77]. Therefore, an increase (rather than a decrease) in serum tryptophan, along with a decrease in kynurenine/tryptophan ratio, may be expected in early allergic inflammation [78]. In fact, Ciprandi et al. (2010) reported that the serum tryptophan concentration is higher among individuals with allergic rhinitis as compared to healthy controls [79]. In addition, serotonin levels have been found to be elevated in the bronchoalveolar lavage fluid of individuals with asthma after allergen provocation [80], which is likely due to serotonin released from mast cells upon degranulation [81]. However, allergen sensitization exerts long-term influences on IDO-1 expression and activity toward developing feedback immune tolerance [82]. For example, IDO-1 is overexpressed in monocytes stimulated by high-affinity receptor for IgE (FcεRI) [83]. Similarly, in individuals with allergic rhinitis, IDO activity is increased in asymptomatic atopics as compared with either symptomatic atopic or nonatopic individuals [84]. This may lead to overactivation of the kynurenine pathway and shift in tryptophan catabolism from serotonin to kynurenine and its metabolites. More research is needed to uncover how allergic inflammation alters levels of serotonin or its metabolite 5-hydroxyindoleacetic acid (5-HIAA) in blood or CSF of individuals with sensitivity to allergens and depression.

Finding the link between allergy and depression also has implications on treatment choices since some medications such as antihistamines can improve allergic symptoms without suppressing the production of inflammatory mediators capable to reach the brain [27], whereas, other medications such as intra-nasal corticosteroids also suppress inflammatory cytokine production. Consistent with this hypothesis, Woo et al. (2011) reported pharmacoecologically that higher numbers of prescriptions of intra-nasal corticosteroids are associated with lower suicide rates at a county level, while higher numbers of prescriptions of antihistamines are associated with a modestly greater suicide risk [85]. Similarly, a recent meta-analysis reported that anti-cytokine therapy (adalimumab, etanercept, infliximab and tocilizumab) in chronic inflammatory conditions also improved depressive symptoms as compared to placebo [86]. More research is needed to fully elucidate the role of anti-inflammatory interventions in depressive disorders.

We found a lower prevalence of S-SAD (0.4%) in the Old Order Amish as compared with other studies that have reported a prevalence up to 9% [18, 19, 21, 22, 87]. It is likely that multiple factors contribute to the low prevalence of S-SAD found in our study. For example, Amish spend more time outdoors and do not have home air conditioning, which may protect individuals from light deprivation associated with spending time in cooler microenvironments in summer. As hypothesized in other populations such as Icelanders and Lapps [88–90], genetic factors may also play a role in low prevalence of S-SAD in Old Order Amish. In addition, the Amish may be more stoical than other populations and may be hesitant to report emotional problems, especially on pen and paper questionnaire completion. However, a sizable proportion of Amish did report a spring/summer pattern of seasonality. It may appear that the Amish may acknowledge changes in mood and behavior relative to summer, and yet not find it problematic. With problem severity not perceived or acknowledged, summer changes of mood and behavior do not reach the threshold for SAD (syndromal or subsyndromal) and this may be another reason for lower prevalence of S-SAD or subsyndromal S-SAD in the Old Order Amish.

The present study also raises a concern about forecasted climate change, which is expected to increase aeroallergen exposure. It has been reported that aeroallergen exposure has increased over the past several decades [91]. Ziska et al. (2011) reported that the duration of the ragweed (*Ambrosia* spp.) pollen season has been increasing in recent decades as a function of latitude in North America since 1995 [92]. Similarly, the average peak value and an annual total of daily counts of airborne pollen have increased by 42.4% and 46.0%, respectively [93]. Interestingly, depression also increased significantly, yet more modestly, from 6.6 % to 7.3%, among persons in the U.S. from 2005 to 2015 [94]. Among adolescents, the prevalence of depression increased from 8.7% in 2005 to 12.7% in 2015 [94]. Suicide rates in the United States have risen nearly 30% since 1999 [95]. Although the exact mechanisms have not been fully elucidated, it is worth considering the potential contribution of the associations among aeroallergen exposure, mood disorders, and suicide.

One major limitation of this study is that it is based on self-report and no data on pollen count were collected. Participants were not asked about history of seasonal allergies or psychiatric illness. We also did not collect longitudinal depression scores for individuals. Another limitation may be the likely overestimation of the rates of SAD by SPAQ in comparison to standard clinical interviews [96, 97]. SPAQ is a research and screening questionnaire that is vulnerable to selection and recall bias. In addition, the study was conducted among the Old Order Amish who are a rural and agrarian population with a distinct lifestyle. While the effect size might have increased due to exposure to the outdoor environment and lower heterogeneity, the generalizability of the findings of this study is likely limited. However, one major strength of this study is that OOA represent a naturalistic population where the confounding effect of electric lighting, substance abuse, and urbanization is negligible. Exposure to aversive elements of the summer season could have been enhanced by the farming activities and the need to keep windows open on hot days, given the absence of air conditioning. The possibility of acquiring tolerance to aeroallergen exposure through daily exposure to aeroallergens during development and throughout adulthood, in particular to agricultural dust containing lipopolysaccharide (LPS), thought to be responsible for the lower prevalence of allergic disease in the Amish [98], do not seem to diminish the association between seasonal summer patterns and mood sensitivity to high pollen counts.

## Conclusion

In conclusion, our results confirm the hypothesis of an association with mood sensitivity to aeroallergens and a summer pattern of seasonality. Also, there was specificity in regard to spring/summer-type SAD and not fall/winter-type SAD manifesting the significant associations (uncorrected for multiple comparisons). This may have potential public health importance considering the high prevalence of allergic rhinitis and asthma [99], and the expected increase in aeroallergen exposure due to forecasted climate change [100].

## List of Abbreviations

BDI-II: Beck Depression Inventory (BDI-) II
IDO-1: Indoleamine-2,3-dioxygenase-1
OOA: Old Order Amish
SAD: Seasonal Affective Disorder
S-SAD: Summer/spring-type Seasonal Affective Disorder
W-SAD: **Fall**/winter-type Seasonal Affective Disorder
ANCOVA: Analysis of Co-Variance
SPAQ: Seasonal Pattern Assessment Questionnaire
